# Haplotype Analysis of Candidate Genes Involved in Inflammation and Oxidative Stress and the Susceptibility to Preeclampsia

**DOI:** 10.1155/2020/4683798

**Published:** 2020-02-26

**Authors:** Aiping Chen, Huifang Zhao, Jingli Wang, Ru Zhang, Jingjing Liu, Xin Zhao, Congying Li, Xuewen Jia, Xueying Li, Yan Lin, Mingzhen Guo, Sai Li, Chao Liu, Yuan Li, Shiguo Liu

**Affiliations:** ^1^Department of Gynecology, The Affiliated Hospital of Qingdao University, Qingdao, China; ^2^School of Nursing, Weifang University of Science and Technology, Shouguang, China; ^3^Medical Genetic Department, Prenatal Diagnosis Center, The Affiliated Hospital of Qingdao University, Qingdao, China; ^4^Department of Laboratory, Qingdao Municipal Hospital, Qingdao, China; ^5^Department of Gynecology and Obstetrics, Qingdao Women and Children's Hospital of Qingdao University, Qingdao, China; ^6^Gynecology, Heze Municipal Hospital, China; ^7^Department of Emergency, Shengli Oilfield Central Hospital, China; ^8^School of Nursing, Binzhou Polytechnic, Binzhou, China; ^9^Clinical Laboratory, The Affiliated Hospital of Qingdao University, Qingdao, China; ^10^Medical Laboratory, Qingdao Women and Children's Hospital of Qingdao University, Qingdao, China; ^11^Biology Teaching and Research Department, Fourth Middle School of Qingdao Economic and Technological Development Zone, Qingdao, China; ^12^Department of Basic Medicine, College of Pharmacy, Jining Medical University, Rizhao, China; ^13^Department of Radiology, Peking Union Medical College Hospital, Chinese Academy of Medical Sciences & Peking Union Medical College, Beijing, China

## Abstract

Unbalanced inflammatory reactions and oxidative stress are inseparably interconnected, and both may play crucial roles in the pathophysiological mechanisms of preeclampsia (PE). In the published previous studies, we have genotyped for SNPs that related to inflammation (rs2227485, rs153109, rs17855750, rs2027432, rs2275913, rs763780, rs4819554, and rs13015714) and oxidative stress (rs1695, rs4680, rs1800566, rs4807542, rs713041, rs7579, rs230813, rs1004467, rs3824755, and rs9932581) to investigate whether these polymorphisms were associated with susceptibility to PE in a Chinese Han population. In this present study, we collected these data of experimental and clinical from above studies for haplotype analysis of inflammation-related SNPs in 631 PE patients and 720 normal pregnancy and oxidative stress-related SNPs in 342 PE patients and 457 normal pregnancies for susceptibility to PE. The data of genotype distribution and allele frequency comparisons after correction for multiple comparisons (P/8 or P/10) showed 2 among the 8 candidate inflammation-related SNPs have significant differences (rs2027432 genotype *χ*^2^ = 407.377, *p* < 0.001, *p* < 0.00625). Moreover, the minor alleles of rs2027432 T (minor allele *χ*^2^ = 450.923, *p* < 0.001, *p* < 0.00625; OR = 21.439, 95%CI = 15.181‐30.278) and rs4819554 G (minor allele *χ*^2^ = 163.465, *p* < 0.001, *p* < 0.00625; OR = 5.814, 95%CI = 4.380‐7.719) were confirmed as risk allele of PE, respectively. Our analysis revealed rs2027432 (TT) of NLRP3 and rs4819554 (GG) of IL-17RA are risk factors for PE. However, no significant difference was found at the oxidative stress-related SNPs. In the candidate loci for oxidative stress, we also identified 3 SNP matches (rs4807542 and rs713041, rs230813 and rs75799, rs1004467 and rs3824755) that had high linkage disequilibrium (LD) with each other and were selected as a block (*r*^2^ = 0.98, *r*^2^ = 0.97, *r*^2^ = 0.97, *r*^2^ > 0.9), and the GT and GC haplotypes of rs4807542 and rs713041 in *GPX4* showed significant differences between the PE and control groups (*χ*^2^ = 5.143, *p* = 0.0233, *p* < 0.05; *χ*^2^ = 6.373, *p* = 0.0116, *p* < 0.05). So, we inferred that polymorphisms of NLRP3 rs2027432 and IL-17RA rs4819554, which are related to inflammation, and the rs713041 variant of *GPX4*, which is related to oxidative stress, were associated with susceptibility to PE. The GT and GC haplotypes of rs4807542 and rs713041 in *GPX4* may increase the risk of PE in the Chinese Han population.

## 1. Introduction

Preeclampsia (PE) is a serious complication of pregnancy characterized by hypertension and proteinuria after 20 weeks of gestation [1]. It can be accompanied by abnormal changes in the heart, lung, liver, kidney, and other vital organs or the blood system, digestive system, nervous system, and placenta-fetal interface, and thus is a major cause of morbidity and mortality in maternal and fetal medicine [2, 3]. The incidence of PE is estimated to be 3-5% of pregnancies worldwide [4], but 8.1% in developing countries, which the mortality rate for mothers can reach 22.0% [5]. The clinical symptoms of PE are reflected in three aspects; the first involves placental perfusion dysfunction followed by a systemic inflammatory response, the second is vascular endothelial damage, and the last is oxidative stress [1]. These placental factors are released into the maternal body and cause the clinical symptoms of PE [6]. Although studies in recent years have suggested that oxidative stress, inflammatory stimuli, vascular endothelial dysfunction, the immune response, and genetic susceptibility are involved in the development and progression of PE [7, 8], the etiology and mechanism remain elusive.

Th1/Th2 immune status keeps in a steady immune status and plays an important role in normal pregnancy [9]. Th2 cells underlie immune responses mediated by interleukin- (IL-) 4, IL-5, IL-13, and IL-10, whereas Th1 cells are involved in the inflammatory response through interferon-*γ* (IFN-*γ*) and IL-2 [10]. Th1 cytokines IL-2, TNF-*α*, and IFN-*γ* are significantly increased, while Th2 cytokines IL-4 and IL-10 are significantly reduced PE patients [11]. Obviously, T lymphocytes are inclined toward Th1 cells and produce an increase in Th1 cytokines and a decrease in Th2 cytokines in PE [9, 12]. This unbalanced immunotolerance causes inflammatory cells to be overactive, adheres to the vascular endothelium, and releases inflammatory factors, such as IL family members and the inflammasome, which eventually abnormally remodels the vascular endothelium to cause PE. Overactivation of Th1 cells after combination of IL-33 and IL-1 not only increase the inflammatory response mediated by Th1 but also induce Th1 cells to release IL-12. IL-12 synergizes with IL-27 to induce native CD4 T^+^ cells to produce increased IFN-*γ*, which leads to the occurrence of PE [13–15]. In addition, IL-1, bound by the IL-1 receptor family member ST2, initiates NF-*κ*B signaling [16], in which NLRP3, as the core of the inflammatory reaction, plays important roles in the development of PE [17]. Fu et al. indicated that uncontrolled Th17 cells can expand the role of inflammation and tissue damage mediators via IL-17 and IL-22 in PE [18].

Normally, the effect of reactive oxygen species (ROS) can be counteracted by antioxidants, such as glutathione and enzymes, including glutathione S-transferases (GSTs), glutathione peroxidases (GPXs), and cytochrome b-245 alpha chain (CYBA) [19, 20]. Oxidative stress is defined as an imbalance between oxidants and antioxidants in the body in which oxidation is more prone to occur and may be involved in the development of PE [7]. Oxidative stress can also participate in the NF-*κ*B pathway and release inflammatory factors and adhesion molecules, leading to the occurrence of PE [21].

Because genetic factors are involved in the development of PE, in this study, we examined single-nucleotide polymorphisms (SNPs) and haplotypes in inflammation- and oxidative stress-associated candidate genes (inflammation genes *IL-22*, *IL-27*, *NLRP3*, *IL-17*, and *IL-1*; oxidative stress genes *GSTP1*, *GPX*, *COMT*, *NQO1*, *SEEP1*, *CYP17*, and *CYBA*) for susceptibility to PE in a Chinese Han population based on our previous study.

## 2. Materials and Methods

### 2.1. Study Population

All PE patients were diagnosed according to guidelines (2015) [22]. The exclusion criteria consisted of chronic hypertension, fetal death, multiple pregnancies, uterine malformation, placental abruption, infection, cancer, in vitro fertilization treatment, gestational diabetes mellitus (GDM), and renal disease or any other potential risk factors for hypertension, including rheumatoid arthritis (RA) and systemic lupus erythematosus (SLE). The control group included women who had no clinical history of PE, with full-term pregnancies and without multiple births, fetal disorder, or any other pathological states. In this present study, a total of 973 patients and 1177 controls were selected from our previous study. That is to say we collected these data of experimental and clinical for the same subjects based on our previous studies for genetic analysis of inflammation-related SNPs in 631 PE patients and 720 normal pregnancy and oxidative stress-related SNPs in 342 PE patients and 457 normal pregnancy for susceptibility to PE in Chinese Han women. The research project was approved by the Ethics Committee of the Affiliated Hospital of Qingdao University.

### 2.2. PCR Amplification/Genotyping

DNA was extracted from peripheral venous blood samples and stored at -20°C. Genotyping of 8 and 10 candidate SNPs related to inflammatory and oxidative stress, respectively, (Tables 1 and 2) was performed using predesigned TaqMan allelic discrimination real-time PCR followed by partial validation by Sanger sequencing. All women who were genotyped were retrospectively confirmed from our previous studies [23–34].

### 2.3. Haplotype Analysis

Haplotype analysis was predicted from genotype data by the computer program Haploview 4.2. Only women with all SNPs successfully genotyped were included in the haplotype analysis (*n*_inflammation_ = 799; *n*_case_ = 342, *n*_controls_ = 457; *n*_oxidative stress_ = 1351; *n*_case_ = 631, *n*_controls_ = 720).

### 2.4. Statistical Analysis

All statistical analyses were performed with the statistical software package SPSS 20 (IBM SPSS Statistics 20). The chi-square test was used to calculate genotypic and allelic frequencies and evaluate the Hardy-Weinberg equilibrium (HWE) in the control group to confirm genetic equilibrium. The relative risk is indicated by the ORs and 95% CIs. In Tables 1 and 2 genotype distribution and allele frequency comparisons, the statistical significance after correction for multiple comparisons (P/8 or P/10) is set at *p* < 0.00625 or *p* < 0.005. Other than that, statistical significance was set at *p* < 0.05. Additionally, linkage disequilibrium blocks and haplotype association risk analyses were conducted using the Haploview 4.2 program.

## 3. Results

### 3.1. Demographic and Clinical Characteristics of the Study Population

The demographic and clinical characteristics of the PE cohort and normal pregnant women in the inflammation and oxidative stress groups are shown in Table 3. No difference was observed in maternal age, gravidity, or number of abortions among the PE and control groups (all *p* > 0.05). The PE group had a higher prevalence of preterm birth, and the gestational age at delivery was lower than that of the control group (*p* < 0.001). The birth weight of newborns in the PE group was lower than that of the control group (*p* < 0.001). The systolic and diastolic blood pressure values of the PE group were significantly higher than those of the control group (*p* < 0.001). As shown in Table 3, the number of white blood cells in the PE group was significantly higher than that in the control group (*p* < 0.001). In the inflammatory group, the PE group had higher neutrophil counts than the control group (*p* = 0.015); however, no significant difference was found in the neutrophil counts for the oxidative stress group (*p* = 0.130).

### 3.2. The Distribution of Genotypes and Allele Frequency

The collected control samples in the study conformed to HWE (rs2227485: *χ*^2^ = 1.500, *p* = 0.221; rs153109 : *χ*^2^ = 0.104, *p* = 0.747; rs17855750 : *χ*^2^ = 0.830, *p* = 0.362; rs2027432 : *χ*^2^ = 0.934, *p* = 0.334; rs2275913 : *χ*^2^ = 0.435, *p* = 0.510; rs763780 : *χ*^2^ = 1.004, *p* = 0.316; rs4819554 : *χ*^2^ = 0.342, *p* = 0.559; rs13015714 : *χ*^2^ = 0.154, *p* = 0.695; rs1695 : *χ*^2^ = 0.121, *p* = 0.728; rs4680 : *χ*^2^ = 0.261, *p* = 0.609; rs1800566 : *χ*^2^ = 2.455, *p* = 0.117; rs4807542 : *χ*^2^ = 0.683, *p* = 0.409; rs713041 : *χ*^2^ = 0.002, *p* = 0.968; rs7579 : *χ*^2^ = 1.702, *p* = 0.192; rs230813 : *χ*^2^ = 0.684, *p* = 0.408; rs1004467 : *χ*^2^ = 0.253, *p* = 0.615; rs3824755 : *χ*^2^ = 0.275, *p* = 0.600; rs9932581 : *χ*^2^ = 0.067, *p* = 0.795). The distributions of the genotypic and allelic frequencies are shown in Tables 1 and 2.

Table 1 shows the inflammation-related genotype distribution and allele frequencies in the PE and control groups. Significant differences were observed for the 2 SNPs among the PE and control groups (rs2027432 genotype *χ*^2^ = 407.377, *p* ≤ 0.00625). Moreover, the minor alleles of rs2027432 T (minor allele *χ*^2^ = 450.923, *p* < 0.001, *p* < 0.00625; OR = 21.439, 95%CI = 15.181‐30.278) and rs4819554 G (minor allele *χ*^2^ = 163.465, *p* < 0.001, *p* < 0.00625; OR = 5.814, 95%CI = 4.380‐7.719) were confirmed as risk alleles for PE. According to Table 1, no significant differences were observed in the remaining candidate SNP loci (rs2227485, rs153109, rs17855750, rs2275913, rs763780, and rs13015714).

For the oxidative stress-related genotype distribution and allele frequencies in the PE and control groups, there was no significant difference at the oxidative stress-related SNPs among 10 groups, although the SNP rs713041 with a C/T polymorphism, the T allele looked like a risk allele for predisposition to PE (minor allele *χ*^2^ = 5.322, *p* = 0.021, *p* < 0.05; OR = 1.196, 95%CI = 1.027‐1.393). Similarly, for all other SNPs in Table 2, there were no significant differences in the remaining SNP loci between PE and control groups.

To further investigate the relationship between the genetic distributions of the PE and control groups, we compared 3 SNPs (rs2027432, rs4819554, rs713041) based on PE classification and staging. First, we divided PE patients into mild and severe PE groups [35].  Table 4 shows the genetic distributions of the mild/severe PE and control groups. The results showed a significant difference in the genetic distribution of rs2027432 in *NLRP3* among the mild/severe PE and control groups (mild PE vs. control: for genotype, *p* < 0.001, for allele, *χ*^2^ = 101.849, *p* < 0.001, OR = 31.959, 95%CI = 11.655‐87.636; severe PE vs. control: for genotype, *p* < 0.001, for allele, *χ*^2^ = 395.685, *p* < 0.001, OR = 20.270, 95%CI = 14.117‐29.106). However, no significant differences were found in another candidate SNP, rs4819554 in *IL17RA* (mild PE vs. control: for genotype, *p* = 0.283, for allele, *χ*^2^ = 2.411, *p* = 0.121, OR = 0.722, 95%CI = 0.477‐1.091; severe PE vs. control: *p* = 0.808, for allele, *χ*^2^ = 0.053, *p* = 0.818, OR = 0.976, 95%CI = 0.791‐1.203).  Table 4 shows a strong association of the genetic distribution of rs713041 in *GPX4* between the severe PE and control groups (for allele, *χ*^2^ = 5.198, *p* = 0.023, OR = 1.210, 95%CI = 1.027‐1.425).

Early-onset PE was diagnosed before 34 weeks of gestation, and PE diagnosed at or after 34 weeks of gestation was considered late-onset PE [36].  Table 5 shows a strong association in the genetic distribution of rs2027432 in *NLRP3* between the early-onset PE and control groups and between the late-onset PE and control groups (early-onset PE vs. control: for genotype, *p* < 0.001, for allele, *χ*^2^ = 294.95, *p* < 0.001, OR = 24.881, 95%CI = 15.399‐40.199; late-onset PE vs. control: *p* < 0.001, for allele, *χ*^2^ = 135.044, *p* < 0.001, OR = 10.335, 95%CI = 6.512‐16.402), whereas no significant differences were found in another candidate SNP, rs4819554 in *IL17RA* (early-onset PE vs. control: for genotype, *p* = 0.315, for allele, *χ*^2^ = 0.494, *p* = 0.482, OR = 1.096, 95%CI = 0.849‐1.414; late-onset PE vs. control: *p* = 0.265, for allele, *χ*^2^ = 0.242, *p* = 0.623, OR = 1.068, 95%CI = 0.822‐1.387).  Table 5 also shows a strong association in the genetic distribution of rs713041 in *GPX4* between the early-onset PE and control groups (for genotype, *p* = 0.018, for allele, *χ*^2^ = 7.280, *p* = 0.007, OR = 1.329, 95%CI = 1.081‐1.636).

### 3.3. LD and Haplotype Analysis

**Figure 1:**
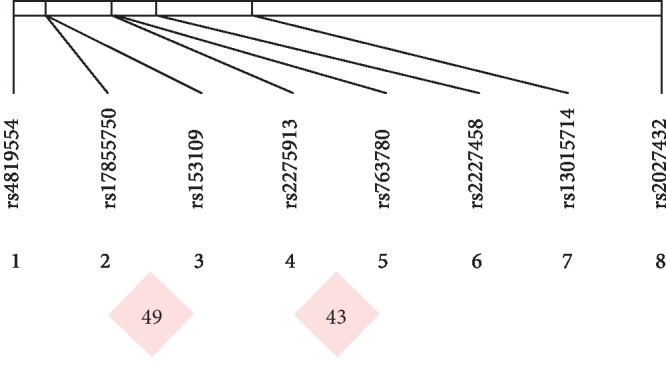
Inflammation-LD plot. We estimated the LD and haplotype using Haploview 4.2; rs153109 and rs17855750 (*IL-27*) and rs2275913 and rs763780 (*IL-17*) were in low LD with each other (*r*^2^ = 0.49 and *r*^2^ = 0.43, *r*^2^ < 0.8).

To further examine the association of the candidate SNPs between the PE and control groups, we estimated the LD and haplotype using Haploview 4.2; rs153109 and rs17855750 (*IL-27*) and rs2275913 and rs763780 (*IL-17*) were in low LD with each other (*r*^2^ = 0.49 and *r*^2^ = 0.43, *r*^2^ < 0.8, Figure 1. Inflammation-LD plot).

**Figure 2:**
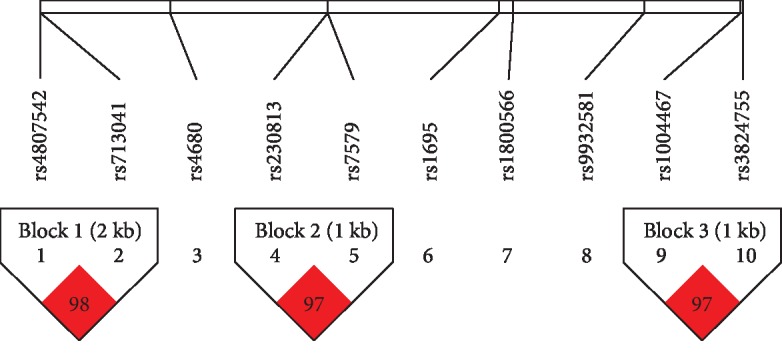
Oxidative stress-LD plot. We estimated the LD and haplotype using Haploview 4.2. Three SNPs had strong correlations; rs4807542 and rs713041(*GPX4*), rs230813 and rs75799 (*SEPP1*), and rs1004467 and rs3824755 (*CYP17A1*) were in high LD with each other and were selected as a block (*r*^2^ = 0.98, *r*^2^ = 0.97, *r*^2^ = 0.97, *r*^2^ > 0.9, *t*). rs713041 in *GPX4* exhibited high LD (*r*^2^ = 0.98) with rs4807542, and both were significantly associated with PE.

We also estimated the LD and haplotype of the oxidative stress-related candidate SNPs. Three SNPs had strong correlations; rs4807542 and rs713041 (*GPX4*), rs230813 and rs75799 (*SEPP1*), and rs1004467 and rs3824755 (*CYP17A1*) were in high LD with each other and were selected as a block (*r*^2^ = 0.98, *r*^2^ = 0.97, *r*^2^ = 0.97, *r*^2^ > 0.9, Figure 2. Oxidative stress-LD plot). rs713041 in *GPX4* exhibited high LD (*r*^2^ = 0.98) with rs4807542, and both were significantly associated with PE.  Table 6 shows the haplotype associations of oxidative stress-related SNPs between the PE and control groups, and two polymorphisms were found (rs4807542/rs713041), indicating that the two primary haplotypes were significantly different. The GT and GC haplotypes from block 1 exhibited the following distribution: 44.6% GT (rs4807542/rs713041), 44.3% GC, and 11% AC. Significant differences in the GT and GC haplotypes were found between the PE and control groups (*χ*^2^ = 5.143, *p* = 0.0233, *p* < 0.05; *χ*^2^ = 6.373, *p* = 0.0116, *p* < 0.05), whereas no differences were found for the remaining haplotypes.

## 4. Discussion

PE is one of the most common and severe obstetric complications and is characterized by a state of excessive inflammatory and oxidative stress. In the second trimester of pregnancy, Th cells play a significant role in the development of PE [10]. Previous studies have indicated that PE is an excessive inflammatory response and associated with a Th1/Th2 immune imbalance in the maternal body. Normal pregnancy associated with a mild inflammatory Th2-based state favors the maternal and fetal environment. In contrast, PE is a proinflammatory state characterized by a Th1-based state [10, 14]. Many studies also suggested that PE may be due to an increase in Th17 cells and a decrease in Treg cells [10, 37–39]. As the core of the inflammatory response, NLRP3 can be activated by many danger signals to exert an immune response to promote the development of PE [17]. IL-27 regulates the differentiation of T cells in the initial stage and plays a crucial role in promoting T1 differentiation and enhancing the activity of T1 cells [40]. Moreover, IL-27 can inhibit the differentiation of Th17 cells by inhibiting the polarization of naive CD4^+^ T cells [41]; in this case, IL-27 induces a variety of biological activities, which may cause the inflammatory response to induce the occurrence of PE [18, 42]. IL-27 promotes Th1 cell differentiation and activity by participating in Th initial differentiation [40] and inhibits Th2 and Th17 activation [41, 43]. The imbalance of Th1/Th2 and Th17/Treg may lead to maternal in the PE susceptibility state. The most important role of IL-17 is to amplify the inflammatory reaction of small vascular endothelial cells, which damages vascular endothelial cells, increases the permeability of blood vessels, and results in the release of a large number of oxygen free radicals. IL-22 combined with IL-22R to activate the JAK1 (mobile kinase IL22R1), Tyk2 (mobile kinase IL10R2), and multiple biological pathways, such as AKT, P38, JNK, and ERK1/2, by phosphorylation of serine and tyrosine in STAT 1, 3, and 5, which finally keeps immune homeostasis [44]. A research found the higher level of IL-22 in the PE mother and newborn cord blood compared with controls [45].

Normally, the effect of ROS can be counteracted by antioxidants, including glutathione and enzymes, such as GSTs, GPXs, CYBA, NQO1, SEEP1, and superoxide dismutase [19, 20]. As a selenoprotein, GPX4 exhibits high antioxidant activity in the body to repress the development of oxidative stress, which promotes the development of PE [46]. In addition, polymorphisms of *GPX4* may affect the expression and antioxidant activity of GPX4 [47]. COMT plays a crucial role in the degradation of both catecholamines and estrogens [48]. During oxidative stress which is an imbalance between oxidants and antioxidants in the body, oxidation is favored, leading to increased inflammatory infiltration and protease secretion. Additionally, activation of the inflammatory response and oxidative stress in the placenta is closely related to the occurrence of PE.

As PE is a complex multigene hereditary disease that was not only associated with many cytokine candidate genes, such as *IL-1* [32, 49], *IL-17*, *IL-22* [39], *NLRP1* [50], and vascular-associated genes [51] but also associated with many oxidative stress genes such as *GSTs*, *GPXs*, *CYBA*, *NQO1*, and *SEEP1* [46, 52–58]. In the published previous studies, we have genotyped for SNPs that related to inflammation (rs2227485, rs153109, rs17855750, rs2027432, rs2275913, rs763780, rs4819554, and rs13015714) and oxidative stress (rs1695, rs4680, rs1800566, rs4807542, rs713041, rs7579, rs230813, rs1004467, rs3824755, and rs9932581) to investigate whether these polymorphisms were associated with susceptibility to PE in a Chinese Han population. We found significant difference for the genotype of IL-22 rs2227485 and GPX4 rs713041 associated with the mild, severe, and early-onset PE. Furthermore, the GPX4 rs713041 C allele has the higher risk for pathogenesis of PE [27, 29]. Those that are just single genes without systematic haplotype analysis. Thus, in this present study, we collected these data of experimental and clinical for the same subjects from above studies for genetic contribution and haplotypes of polymorphisms of inflammation-related SNPs in 631 PE patients and 720 normal pregnancy and oxidative stress-related SNPs in 342 PE patients and 457 normal pregnancies for susceptibility to PE in Chinese Han women.

Our study found significant differences in the genetic distributions of rs2027432 in *NLRP3*, rs4819554 in *IL-17RA*, and rs713041 in *GPX4* between the PE and control groups. We further divided the PE group into mild/severe and early/late-onset subgroups and compared them with the control group. Significant differences were also found for rs2027432 in *NLRP3* and rs713041 in *GPX4*; however, no significant difference was found in the subgroup analysis of rs4819554 in *IL-17RA*. Our results suggest that the two SNPs, rs2027432 in *NLRP3* and rs713041 in *GPX4*, may be associated with risks for PE in Chinese Han women.

Analysis of LD showed that rs153109 and rs17855750 in *IL-27* and rs2275913 and rs763780 in *IL-17* were in low LD with each other (*r*^2^ < 0.8, Figure 1. Inflammation-LD plot), indicating that no substitution occurs between them. Unfortunately, no haplotype formation was found in the analysis of inflammatory factors, partly due to an imbalance in the HWE or the scatter position distribution of SNPs. Therefore, such contradictory results suggest that our findings should be validated using large samples that include different countries.

However, we identified 3 SNP matches, rs4807542 and rs713041 (*GPX4*), rs230813 and rs75799 (*SEPP1*), and rs1004467 and rs3824755 (*CYP17A1*), among oxidative stress genes that were in high LD, and significant substitutability with each other was observed. The analysis of haplotype correlation showed that the GT and GC haplotypes in block 1 (rs4807542/rs713041) were significantly different, which suggested that pregnant women carrying the GT and/or GC haplotypes were more likely to suffer from PE. To our knowledge, this is the first study of correlations of inflammation and oxidative stress with PE susceptibility in a Chinese Han population with both an LD and haplotype analysis. However, our findings should be confirmed in individuals of different races and geographic locations. Our previous studies demonstrated that the two SNPs (rs2227485 in *IL-22* and s713041 in *GPX4*) are associated with risks for PE. We found that the rs2227485 in *IL-22* showed a significant difference in the allele for the early-onset PE group and the genotype of the late-onset PE and control subgroups. The GPX4 rs713041 allele C was associated with an increased risk for PE in a previous study. Additionally, the rs713041 genotype was associated with the mild, severe, and early-onset PE. These genes may play a key role in the pathogenesis of PE.

In conclusion, we found that oxidative stress and the inflammatory response may play an inseparable role in the progression of PE, which provides the basis for revealing the genetic mechanism of PE. As few studies have performed a haplotype analysis of candidate genes related to inflammatory cytokines and oxidative stress in PE, further experiments are needed to verify these findings.

## Figures and Tables

**Table 1:** Genotype distribution and allele frequencies of the human inflammation related gene in cases and controls.

		Genotype frequency of cases			Genotype frequency of controls			Genotype frequency		Minor allele	Minor allele frequencies					
SNP ID (allele1/allele2)	Position	1/1	1/2	2/2	1/1	1/2	2/2	*χ* ^2^	*p*		Case	Control	*χ* ^2^	*p*	OR	95% CI
rs2227485 (C/T)	68253933	107	188	47	152	234	71	1.187	0.552	T	0.412	0.411	0.001	0.971	0.996	0.815-1.218
rs153109 (C/T)	28507775	60	154	128	62	217	178	2.399	0.301	C	0.401	0.373	1.250	0.264	1.123	0.916-1.376
rs17855750 (G/T)	28503907	8	64	270	11	104	342	1.957	0.376	G	0.117	0.138	1.521	0.217	0.828	0.614-1.118
rs2027432 (C/T)	247415139	304	36	2	79	234	144	407.377	≤0.001	T	0.058	0.571	450.923	≤0.001	21.439	15.181-30.278
rs2275913 (A/G)	52186235	65	155	122	82	215	160	0.270	0.874	A	0.417	0.415	0.006	0.936	1.008	0.825-1.233
rs763780 (C/T)	52236941	2	59	281	5	68	384	1.353	0.508	C	0.092	0.085	0.223	0.637	1.087	0.768-1.540
rs4819554 (A/G)	17084145	99	181	62	146	230	81	0.848	0.654	G	0.446	0.429	163.465	≤0.001	5.814	4.380-7.719
rs13015714 (G/T)	102355405	103	156	83	125	232	100	2.080	0.353	T	0.471	0.473	0.006	0.940	1.008	0.826-1.229

Table 1 shows the results of inflammation-related genotype distribution and allele frequencies in PE and control groups. After correction for multiple comparisons (P/8), 2 among 8 groups of the candidate inflammation-related SNPs have significant differences (rs2027432 genotype *χ*^2^ = 407.377, *p* < 0.001, *p* < 0.00625). Moreover, the minor allele of rs2027432 T (minor allele *χ*^2^ = 450.923, *p* < 0.001, *p* < 0.00625; OR = 21.439, 95%CI = 15.181‐30.278) and rs4819554 G (minor allele *χ*^2^ = 163.465, *p* < 0.001, *p* < 0.00625; OR = 5.814, 95%CI = 4.380‐7.719) were confirmed as risk allele of PE, respectively. According to Table 1, there were no significant differences in the remaining candidate SNP loci (rs2227485: for genotype, *p* = 0.552, for allele, *χ*^2^ = 0.001, *p* = 0.971, OR = 0.996, 95%CI = 0.815‐1.218; rs153109: for genotype, *p* = 0.301, for allele, *χ*^2^ = 1.250, *p* = 0.264, OR = 1.123, 95%CI = 0.916‐1.376; rs17855750: for genotype, *p* = 0.376, for allele, *χ*^2^ = 1.521, *p* = 0.217, OR = 0.828, 95%CI = 0.614‐1.118; rs2275913: for genotype, *p* = 0.874, for allele, *χ*^2^ = 0.006, *p* = 0.936, OR = 1.008, 95%CI = 0.825‐1.233; rs763780: for genotype, *p* = 0.508, for allele, *χ*^2^ = 0.233, *p* = 0.637, OR = 1.087, 95%CI = 0.768‐1.540; rs4819554: for genotype, *p* = 0.654; rs13015714: for genotype, *p* = 0.353, for allele, *χ*^2^ = 0.006, *p* = 0.940, OR = 1.008, 95%CI = 0.826‐1.229).

**Table 2:** Genotype distribution and allele frequencies of the human oxidative stress-related gene in cases and controls.

		Genotype frequency of cases			Genotype frequency of controls			Genotype frequency		Minor allele	Minor allele frequencies					
SNP ID (allele1/allele2)	Position	1/1	1/2	2/2	1/1	1/2	2/2	*χ* ^2^	*p*		Case	Control	*χ* ^2^	*p*	OR	95% CI
rs1695 (A/G)	67585218	394	214	23	452	235	33	0.885	0.642	G	0.206	0.209	0.037	0.848	1.018	0.845-1.227
rs4680 (A/G)	19963748	48	227	356	52	273	395	0.120	0.758	A	0.256	0.262	0.120	0.729	0.970	0.816-1.153
rs1800566 (C/T)	69711242	177	330	124	219	337	164	4.239	0.120	C	0.458	0.462	0.039	0.843	1.015	0.873-1.182
rs4807542 (A/G)	1104079	9	118	504	7	149	564	1.363	0.506	A	0.108	0.113	0.201	0.654	0.946	0.743-1.204
rs713041 (C/T)	1106616	216	296	119	204	359	157	5.796	0.055	T	0.423	0.467	5.322	0.021	1.196	1.027-1.393
rs7579 (A/G)	42800706	51	249	331	48	302	370	1.502	0.472	A	0.278	0.276	0.010	0.920	1.009	0.852-1.194
rs230813 (C/G)	42798931	105	276	250	104	353	263	3.914	0.141	C	0.385	0.390	0.057	0.811	0.981	0.840-1.146
rs1004467 (C/T)	102834750	80	279	272	97	326	297	0.522	0.770	C	0.348	0.361	0.516	0.473	0.944	0.806-1.105
rs3824755 (C/G)	102836092	72	278	281	85	334	301	1.029	0.598	C	0.334	0.35	0.728	0.394	0.933	0.796-1.094
rs9932581 (C/T)	88651945	107	337	187	159	355	206	5.714	0.057	C	0.437	0.467	2.567	0.109	0.883	0.759-1.028

Table 2 shows the results of oxidative stress-related genotype distribution and allele frequencies in PE and control groups. After correction for multiple comparisons (P/10), there was no significant difference at the oxidative stress-related SNPs among 10 groups, although the SNP rs713041 with a C/T polymorphism, the T allele looks like a risk allele for predisposition to PE (minor allele *χ*^2^ = 5.322, *p* = 0.021, *p* < 0.05; OR = 1.196, 95%CI = 1.027‐1.393). Similarly, for all other SNPs in Table 2, there were no significant differences in the remaining SNP loci between PE and control groups (rs1695: for genotype, *p* = 0.642, for allele, *χ*^2^ = 0.037, *p* = 0.848, OR = 1.018, 95%CI = 0.845‐1.227; rs4680: for genotype, *p* = 0.758, for allele, *χ*^2^ = 0.120, *p* = 0.729, OR = 0.970, 95%CI = 0.816‐1.153; rs1800566: for genotype, *p* = 0.120, for allele, *χ*^2^ = 0.039, *p* = 0.843, OR = 1.015, 95%CI = 0.873‐1.182; rs4807542: for genotype, *p* = 0.506, for allele, *χ*^2^ = 0.201, *p* = 0.654, OR = 0.946, 95%CI = 0.743‐1.204; rs713041: for genotype, *p* = 0.055; rs7579: for genotype, *p* = 0.472, for allele, *χ*^2^ = 0.010, *p* = 0.920, OR = 1.009, 95%CI = 0.852‐1.194; rs230813: for genotype, *p* = 0.141, for allele, *χ*^2^ = 0.057, *p* = 0.811, OR = 0.981, 95%CI = 0.840‐1.146; rs1004467: for genotype, *p* = 0.770, for allele, *χ*^2^ = 0.516, *p* = 0.473, OR = 0.944, 95%CI = 0.806‐1.105; rs3824755: for genotype, *p* = 0.598, for allele, *χ*^2^ = 0.728, *p* = 0.394, OR = 0.933, 95%CI = 0.796‐1.094; rs9932581: for genotype, *p* = 0.057, for allele, *χ*^2^ = 2.567, *p* = 0.109, OR = 0.883, 95%CI = 0.759‐1.028).

**Table 3:** Demographic and clinical characteristics of the inflammation/oxidative stress (1/2) groups.

Characteristics	PE (342/631)	Controls (457/720)	*T*	*p* value
	1/2	1/2	1 vs. 1/2 vs. 2	1 vs. 1/2 vs. 2
Maternal age (years)	30.45 ± 5.82/30.23 ± 5.59	30.80 ± 4.52/30.15 ± 3.92	-0.930/0.295	0.353/0.768
Gravidity (times)	2.25 ± 1.21/2.25 ± 1.27	2, 38 ± 1.24/2.19 ± 1.08	-1.422/1.001	0.156/0.317
Abortion (times)	0.67 ± 0.91/0.62 ± 0.93	0.75 ± 0.94/0.62 ± 0.80	-1.290/0.016	0.197/0.987
Gestational age (weeks)	34.59 ± 3.81/35.00 ± 3.67	38.71 ± 1.60/39.15 ± 1.43	-18.812/-26.697	<0.001/<0.001
Gestational age at delivery (weeks)	35.27 ± 3.10/35.56 ± 3.32	39.01 ± 1.32/39.45 ± 1.11	-20.928/-28.067	<0.001/<0.001
Birth weight (kg)	2.38 ± 0.90/2.48 ± 0.92	3.38 ± 0.35/3.43 ± 0.34	-19.467/-24.676	<0.001/<0.001
Systolic blood pressure (mmHg)	161.49 ± 19.48/160.74 ± 19.22	113.78 ± 10.29/114.24 ± 9.36	41.197/55.286	<0.001/<0.001
Diastolic blood pressure (mmHg)	105.06 ± 14.32/104.97 ± 14.05	74.19 ± 7.87/73.29 ± 7.84	36.004/50.194	<0.001/<0.001
White blood cells (×10^9^/L)	9.80 ± 2.95/9.79 ± 3.86	8.87 ± 2.49/7.33 ± 3.78	4.733/4.421	<0.001/<0.001
Neutrophil (×10^9^/L)	7.34 ± 2.69/8.99 ± 2.51	6.76 ± 3.71/7.03 ± 3.29	2.433/1.515	0.015/0.130

**Table 4:** The comparison of genetic distributions between mild/severe PE and control groups.

Group	*N* I/II	rs2027432 (I)					rs4819554 (I)					rs713041 (II)				
		CC	CT	TT	C	T	AA	AG	GG	A	G	CC	CT	TT	C	T
Mild PE	50/141	46	4	0	96	4	11	27	12	49	51	47	66	28	160	122
Control	457/720	79	234	144	392	522	146	230	81	522	392	204	359	157	767	673
*χ* ^2^		135.716			101.849		2.528			2.411		1.442			1.145	
*p*		<0.001			<0.001		0.283			0.121		0.486			0.285	
OR					31.959					0.722					1.151	
95% CI					11.655-87.636					0.477-1.091					0.890-1.488	
Severe PE	292/490	258	32	2	548	36	88	154	50	330	254	169	230	91	568	412
Control	457/720	79	234	144	392	522	146	230	81	522	392	204	359	157	767	673
*χ* ^2^		368.100			395.685		0.426			0.053		5.584			5.198	
*p*		<0.001			<0.001		0.808			0.818		0.061			0.023	
OR					20.270					0.976					1.210	
95% CI					14.117-29.106					0.791-1.203					1.027-1.425	

Table 4 shows the results of genetic distributions between mild/severe PE and control groups. The results show rs2027432 in NLRP3 significant difference between mild/severe PE and control groups in the genetic distributions (mild PE vs. control: for genotype, *p* < 0.001, for allele, *χ*^2^ = 101.849, *p* < 0.001, OR = 31.959, 95%CI = 11.655‐87.636; severe PE vs. control: for genotype, *p* < 0.001, for allele, *χ*^2^ = 395.685, *p* < 0.001, OR = 20.270, 95%CI = 14.117‐29.106). While no significant differences were found in another candidate SNP rs4819554 in *IL17RA* (mild PE vs. control: for genotype, *p* = 0.283, for allele, *χ*^2^ = 2.411, *p* = 0.121, OR = 0.722, 95%CI = 0.477‐1.091; severe PE vs. control: *p* = 0.808, for allele, *χ*^2^ = 0.053, *p* = 0.818, OR = 0.976, 95%CI = 0.791‐1.203). In Table 4, it also showed that there was a strong association in the genetic distributions of rs713041 in *GPX4* between severe PE and control groups (for allele, *χ*^2^ = 5.198, *p* = 0.023, OR = 1.210, 95%CI = 1.027‐1.425).

**Table 5:** The comparison of genetic distributions between early/late-onset PE and control groups.

Group	*N* I/II	rs2027432(I)					rs4819554(I)					rs713041(II)				
		CC	CT	TT	C	T	AA	AG	GG	A	G	CC	CT	TT	C	T
Early-onset PE	187/249	168	19	0	355	19	52	93	42	197	135	94	112	43	300	198
Control	457/720	79	234	144	392	522	146	230	81	522	392	204	359	157	767	673
*χ* ^2^		297.949			294.95		2.307			0.494		8.088			7.280	
*p*		<0.001			<0.001		0.315			0.482		0.018			0.007	
OR					24.881					1.096					1.329	
95% CI					15.399-40.199					0.849-1.414					1.081-1.636	
Late-onset PE	155/382	136	17	2	289	21	47	88	20	182	128	122	184	76	428	336
Control	457/720	79	234	144	392	522	146	230	81	522	392	204	359	157	767	673
*χ* ^2^		253.539			135.044		2.653			0.242		1.672			1.529	
*p*		<0.001			<0.001		0.265			0.623		0.433			0.216	
OR					10.335					1.068					1.118	
95% CI					6.512-16.402					0.822-1.387					0.937-1.333	

Table 5 shows that there existed a strong association in the genetic distributions of rs2027432 in NLRP3 between early-onset PE and control groups, late-onset PE and control groups (early-onset PE vs. control: for genotype, *p* < 0.001, for allele, *χ*^2^ = 294.95, *p* < 0.001, OR = 24.881, 95%CI = 15.399‐40.199; late-onset PE vs. control: *p* < 0.001, for allele, *χ*^2^ = 135.044, *p* < 0.001, OR = 10.335, 95%CI = 6.512‐16.402), while no significant differences were found in another candidate SNP rs4819554 in *IL17RA* (early-onset PE vs. control: for genotype, *p* = 0.315, for allele, *χ*^2^ = 0.494, *p* = 0.482, OR = 1.096, 95%CI = 0.849‐1.414; late-onset PE vs. control: *p* = 0.265, for allele, *χ*^2^ = 0.242, *p* = 0.623, OR = 1.068, 95%CI = 0.822‐1.387). In Table 5, it also showed that there was a strong association in the genetic distributions of rs713041 in *GPX4* between early-onset PE and control groups (for genotype, *p* = 0.018, for allele, *χ*^2^ = 7.280, *p* = 0.007, OR = 1.329, 95%CI = 1.081‐1.636).

**Table 6:** The haplotype associations of oxidative stress-related SNPs between PE and controls.

	Freq.	PE, control ratio counts	PE, control frequencies	Chi-square	*p* value
Block 1					
GT	0.446	534.0 : 728.0, 671.0 : 767.0	0.423, 0.467	5.143	0.0233
GC	0.443	592.0 : 670.0, 605.0 : 833.0	0.469, 0.421	6.373	0.0116
AC	0.11	136.0 : 1126.0, 162.0 : 1276.0	0.108, 0.113	0.164	0.6857
Block 2					
GG	0.608	771.4 : 490.6, 871.2 : 568.8	0.611, 0.605	0.109	0.7414
CA	0.273	346.4 : 915.6, 390.2 : 1049.8	0.274, 0.271	0.041	0.8397
CG	0.115	139.6 : 1122.4, 170.8 : 1269.2	0.111, 0.119	0.418	0.5177
Block 3					
TG	0.639	818.9 : 443.1, 908.9 : 531.1	0.649, 0.631	0.916	0.3385
CC	0.337	417.9 : 844.1, 492.9 : 947.1	0.331, 0.342	0.373	0.5412
CG	0.018	21.1 : 1240.9, 27.1 : 1412.9	0.017, 0.019	0.171	0.6792

Apparently, Table 6 indicates the haplotype associations of oxidative stress-related SNPs between PE and controls; there were two polymorphisms (rs4807542/rs713041) stated two primary haplotypes had significant difference, that is the GT and GC haplotypes from block 1, which produced the following distribution: 44.6% GT (rs4807542/rs713041), 44.3% GC, and 11% AC. Significantly statistical differences were identified in haplotype GT and GC between PE and control groups (*χ*^2^ = 5.143, *p* = 0.0233, *p* < 0.05; *χ*^2^ = 6.373, *p* = 0.0116, *p* < 0.05), while there were no differences in the rest of haplotypes.

## Data Availability

All data used to support the findings of this study are included within the article.
